# ESBL-Producing, Carbapenem- and Ciprofloxacin-Resistant *Escherichia coli* in Belgian and Dutch Broiler and Pig Farms: A Cross-Sectional and Cross-Border Study

**DOI:** 10.3390/antibiotics10080945

**Published:** 2021-08-04

**Authors:** Sien De Koster, Moniek Ringenier, Christine Lammens, Arjan Stegeman, Tijs Tobias, Francisca Velkers, Hans Vernooij, Marjolein Kluytmans-van den Bergh, Jan Kluytmans, Jeroen Dewulf, Herman Goossens, on behalf of the i-4-1-Health Study Group

**Affiliations:** 1 Laboratory of Medical Microbiology, Vaccine and Infectious Diseases Institute, University of Antwerp, 2000 Antwerp, Belgium; sien.dekoster@uantwerpen.be (S.D.K.); christine.lammens@uantwerpen.be (C.L.); 2 Veterinary Epidemiology Unit, Department of Reproduction, Obstetrics and Herd Health, Faculty of Veterinary Medicine, Ghent University, 9820 Merelbeke, Belgium; Moniek.Ringenier@UGent.be (M.R.); jeroen.dewulf@ugent.be (J.D.); 3 Department of Population Health Sciences, Faculty of Veterinary Medicine, Utrecht University, 3584 CL Utrecht, The Netherlands; J.A.Stegeman@uu.nl (A.S.); t.j.tobias@uu.nl (T.T.); F.C.Velkers@uu.nl (F.V.); J.C.M.Vernooij@uu.nl (H.V.); 4 Department of Infection Control, Amphia Hospital, 4818 CK Breda, The Netherlands; marjoleinkluytmans@gmail.com; 5 Julius Center for Health Sciences and Primary Care, UMC Utrecht, University of Utrecht, 3584 CG Utrecht, The Netherlands; jankluytmans@gmail.com; 6 Amphia Academy Infectious Disease Foundation, Amphia Hospital, 4818 CK Breda, The Netherlands; 7 Microvida Laboratory for Microbiology, Amphia Hospital, 4818 CK Breda, The Netherlands

**Keywords:** antibiotic resistance, antibiotic use, *Escherichia coli*, broilers, pigs

## Abstract

Background. The use of antibiotics in food production selects for resistant bacteria and may cause a threat to human and animal health. Belgium and the Netherlands have one of the highest densities of broilers and pigs in Europe, making active monitoring of antibiotic use and resistance in this region vital. Objectives. This study aimed to quantify ESBL-producing (ESBL-*E. coli*), carbapenem- and ciprofloxacin-resistant (CiproR) *Escherichia coli* in animal feces on broiler and pig farms with a history of high antibiotic use in Belgium and the Netherlands. Methods. A total of 779 broiler and 817 pig fecal samples, collected from 29 conventional broiler and 31 multiplier pig farms in the cross-border region of Belgium and the Netherlands, were screened for the presence of antibiotic-resistant *E. coli* using selective culturing. Results. Carbapenem-resistant *E. coli* were not detected. ESBL-*E. coli* were remarkably more prevalent in samples from Belgian than Dutch farms. However, CiproR-*E. coli* were highly prevalent in broilers of both countries. The percentage of samples with ESBL- and CiproR-*E. coli* was lower in pig compared to poultry farms and varied between farms. No clear association with the on-farm antibiotic use in the year preceding sampling was observed. Multidrug resistance was frequently observed in samples from both countries, but ESBL-production in combination with ciprofloxacin resistance was higher in samples from Belgium. Conclusions. This study demonstrated marked differences in antibiotic resistance between countries, farms and within farms. The observed variation cannot be explained straightforward by prior quantity of antibiotic use suggesting that it results from more complex interactions that warrant further investigation.

## 1. Introduction

Pig and poultry meat is often produced in specialized and intensive livestock systems with high animal densities, large production units with application of strict biosecurity measures and use of preventive vaccinations and antibiotic treatments [1]. The use of antibiotics in farm animals may select for bacteria resistant to antibiotics, possibly including those used in human medicine. A considerable amount of applied antibiotic substances ends up in the intestines [2]. Consequently, the gastrointestinal tract of livestock is an important reservoir for the selection of antibiotic resistance. 

Currently, the increasing resistance in Gram-negative enteric bacilli receives special attention because of the potential horizontal spread to pathogens [3,4,5]. In *Escherichia coli,* extended-spectrum beta-lactamase (ESBL)-production and carbapenem and fluoroquinolone resistance result in a decreased efficiency of critically important antibiotics, such as third- and fourth-generation cephalosporins, meropenem and ciprofloxacin [6,7]. Resistance to these substances in intestinal bacteria of animals has become a threat to human health because of the potential risk of spread to humans [8]. Dissemination can occur via direct contact, exposure to feces via agricultural and human waste, fecal contamination of carcasses during slaughter and contaminated food or drinking water [4,9]. Although livestock and food-associated reservoirs are not major contributors to the ESBL occurrence in humans [10], transmission between reservoirs is likely to occur [11,12,13,14].

The south and central parts of the Netherlands and Flanders (Belgium) have one of the highest livestock densities in Europe [15]. Both countries have comparable farming practices [15], yet total antimicrobial use in food-producing animals in Belgium is still relatively high (113.1 mg/population correction unit) compared to the Netherlands (57.5 mg/population correction unit) in 2018 [16]. Overall, in line with the reduction in use [17,18], a reduction in the prevalence of antibiotic resistance in commensal *E. coli* bacteria in animals in the Netherlands [17] and in Belgium is observed [19]. Still, considerable variations in antibiotic use between farms and between countries have been observed for pigs and broilers [20,21,22]. To better understand factors affecting antibiotic resistance and to implement stewardship actions more effectively, understanding antibiotic use and resistance on animal species and farm level in each country is essential. National (farm-level) monitoring systems from distinct countries differ in data collection, analyses, and reporting, making comparison of outcomes difficult. In this study, harmonized and comparable data on antibiotic use and resistance in food-producing animals at farm level in Belgium and the Netherlands is used, providing opportunities to compare antibiotic use and resistance and to study the origin and relevance of these differences. The aim of this study was to investigate the percentage of samples with ESBL-producing *E. coli* (ESBL-*E. coli*), carbapenem-resistant and ciprofloxacin-resistant *E. coli* (CiproR-*E. coli*) in Belgian and Dutch pig and poultry farms with a history of high antibiotic use.

## 2. Results

### 2.1. Antibiotic Use in Belgian and Dutch Broiler and Pig Farms

**Figure 1:**
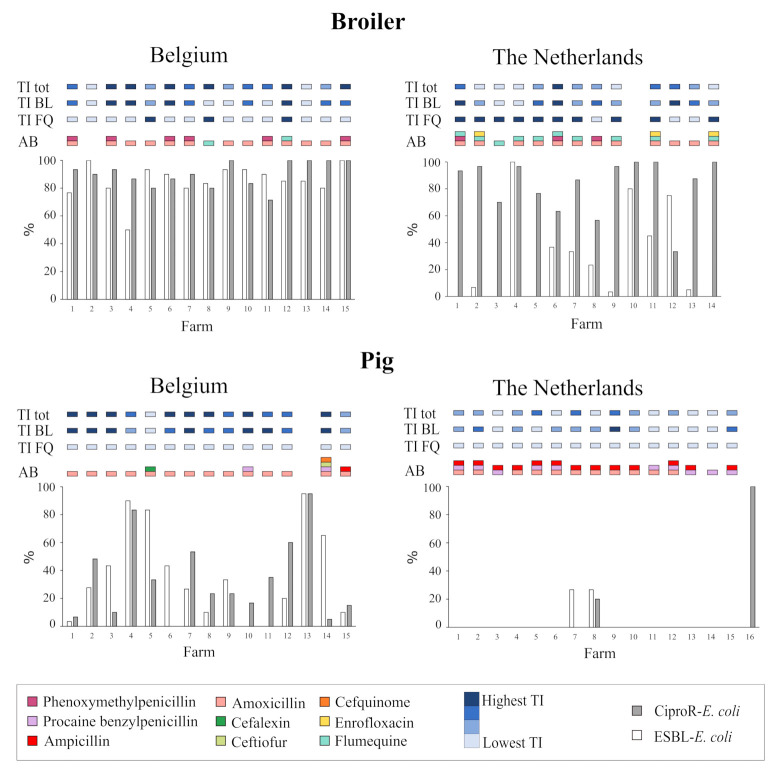
Percentages of ESBL-*E. coli* and CiproR-*E. coli* in Belgian and Dutch broiler and pig farms and the use of antibiotics at farm level. Antibiotic use in the year preceding sampling is presented as treatment incidence (TI) of total antibiotic use (TI tot), beta-lactam (TI BL) and fluoroquinolone (TI FQ) antibiotics. Colors indicate the active substance of the antibiotic (AB) used. Lowest to highest TI was indicated with a blue gradient. The total TI and beta-lactam TI was categorized based on quartiles. The TI of fluoroquinolones was categorized based on use or no use. For Dutch pig farm ID ten, eleven and twelve prevalence of ciprofloxacin-resistant *E. coli* was not determined. For Belgian pig farm ID 13 and Dutch pig farm ID 16, data on antibiotic use was not available for publication.

The total treatment incidence (TI) of beta-lactams and fluoroquinolones and active substances of these antibiotics used one year before sampling per farm are shown in Figure 1 and Supplementary Materials Tables S1 and S2. In the year before sampling, no carbapenems nor third- or fourth-generation cephalosporins were used in the Belgian and Dutch broiler farms. In ten out of fourteen Dutch broiler farms, the active compound flumequine was used, and enrofloxacin was additionally used in three of these farms. In Belgium, two out of fifteen broiler farms used flumequine. Carbapenems, third- and fourth-generation cephalosporins or (fluoro)quinolones were not used in Dutch pig farms in the study period. In the Belgian pig farms, third-generation cephalosporines (ceftiofur and cefquinome) were used in one farm, and no (fluoro)quinolones or carbapenems were used. Beta-lactam antibiotics were prescribed in 92% of the studied farms. In general, the total TI and TI of beta-lactams was higher in Belgium compared to the Netherlands, both in weaned pigs and broilers. The type of beta-lactams prescribed in broilers were the penicillinase-sensitive beta-lactam phenoxymethylpenicillin and the broad-spectrum beta-lactam amoxicillin. In pigs, amoxicillin was frequently used in Belgium, while in the Netherlands, procaine benzylpenicillin, ampicillin and amoxicillin were prescribed.

### 2.2. ESBL-Producing, Carbapenem-Resistant and Ciprofloxacin-Resistant E. coli in Belgian and Dutch Broiler and Pig Farms

A total of 779 broiler and 817 pig fecal samples were tested, covering 89% of the total aimed number of samples. Due to invalid sampling (*n* = 2) and limitation of laboratory materials for selective culturing, the envisaged total number of 1800 samples could not be achieved. Of all resistant bacterial isolates (1855 isolates from 1596 samples), 91.4% were identified as *E. coli*. Other Enterobacteriaceae were present in low numbers, namely *Citrobacter freundii* (0.05%), *Escherichia fergusonii* (0.6%), *Klebsiella pneumoniae* (1.78%), *Morganella morganii* (0.16%), *Proteus* spp. (5.90%) and *Providencia rettgeri* (0.05%), and were excluded from further analysis.

In none of the samples were carbapenem-resistant Enterobacteriaceae detected. In general, the percentage of samples positive for resistant bacteria in pig farms was notably lower compared to broiler farms after selective culturing. In pigs, ESBL-*E. coli* and CiproR-*E. coli* were more prevalent in Belgium than in the Netherlands. In Belgian broilers, the percentage of ESBL-*E. coli* was high compared to Dutch broilers (Table 1). The within-farm percentage of ESBL-*E. coli* was above 70% in 14/15 Belgian broiler farms compared to 3/14 of the broiler farms in the Netherlands (Figure 1). In contrast, the percentage of CiproR-*E. coli* in broilers was high in both countries. All participating broiler farms tested positive for the presence of CiproR-*E. coli* and 26 out of 29 farms showed a percentage of positive samples of 70% or higher after selective culturing of resistant bacteria. The percentage of resistant bacteria varied greatly between farms. Moreover, variations in resistance between different units of the same farm were observed (Supplementary Figure S1).

### 2.3. Associations between Antimicrobial Use and Resistance

No association between the level of antibiotic use and the percentage of resistant samples at farm level in broiler and pig farms was found (Table 2). When studying the association between the total antibiotic use and the percentage of ESBL-*E. coli* and CiproR-*E. coli* positive samples, a lower odds for a positive sample was observed in farms with a higher use compared to farms with the lowest use in this study. One exception was the positive, yet not significant, association between total antibiotic use and the percentage of *E. coli* positive samples in the third quartile category of antibiotic use (OR 1.2). The presence of ESBL-*E. coli* was generally not associated with higher beta-lactam use in farms. In contrast, although not significant, a higher odds for the presence of CiproR-*E. coli* was found in broiler farms that used fluoroquinolones in the year preceding sampling. 

### 2.4. Antibiotic Resistance in ESBL-E. coli and CiproR-E. coli from Broiler Chickens and Pigs

**Figure 2:**
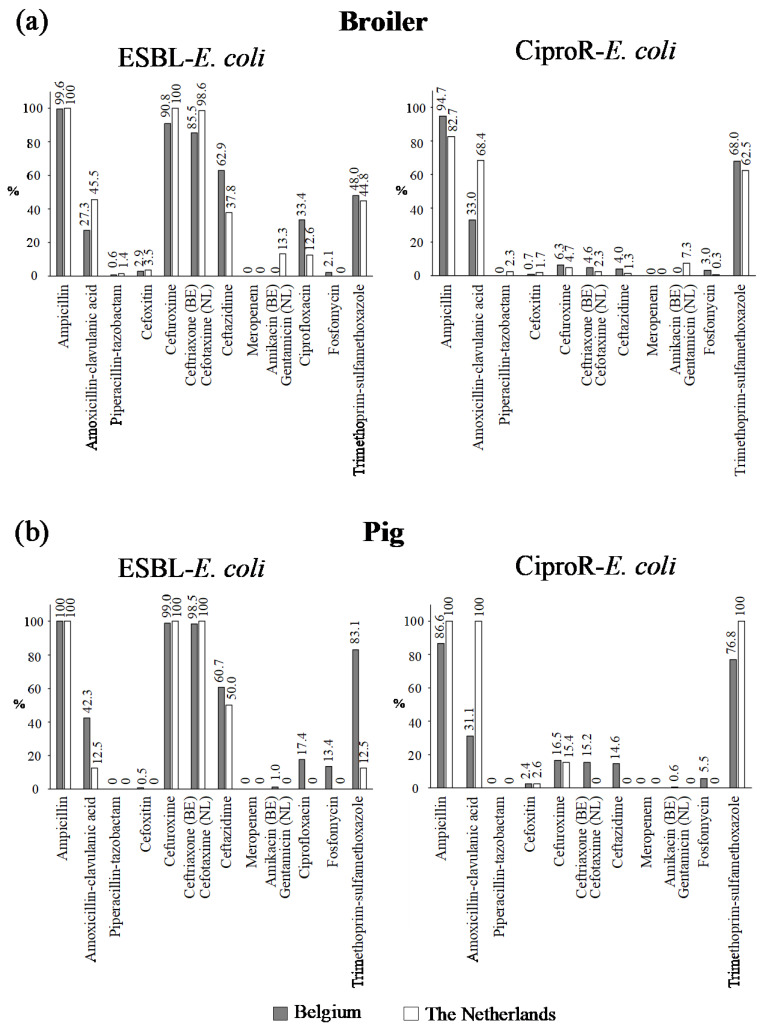
Percentage of antibiotic resistance per type of antibiotic in all ESBL-*E. coli* and CiproR-*E. coli* isolates from broiler chickens (**a**) and weaned pigs (**b**) in Belgium and the Netherlands. Number of ESBL-*E. coli* from broilers: N BE = 523, N NL = 143, number of CiproR-*E. coli* from broilers: N BE = 303, N NL = 301. Number of ESBL-*E. coli* from pigs: N BE = 201, N NL = 16, number of CiproR-*E. coli* from pigs: N BE = 164, N NL = 39.

No meropenem resistance was found in *E. coli* from the feces of broilers and pigs (Figure 2). ESBL-*E. coli* were resistant to ampicillin, cefuroxime and ceftriaxone (BE) or cefotaxime (NL). Resistance to piperacillin–tazobactam, cefoxitin, fosfomycin and amikacin/gentamycin was generally low. In broilers, 33.4% of the Belgian ESBL-*E. coli* were co-resistant to ciprofloxacin, whereas in the Netherlands, 12.6% of the isolates showed ESBL-production in combination with ciprofloxacin resistance. No resistance to ciprofloxacin was found in ESBL-*E. coli* isolates from Dutch pigs. In Belgian pigs, 17.4% of the ESBL-*E. coli* were co-resistant for ciprofloxacin. 

Resistance to ampicillin was high (>80%) in CiproR-*E. coli* in both animal species and both countries. Resistance exclusive to ciprofloxacin was found in 4.0% of the Belgian broilers whereas 14.9% of the Dutch CiproR-*E. coli* from broilers were resistant exclusively to ciprofloxacin. In pigs, this is the case for 6.7% of the Belgian and none of the Dutch CiproR-*E. coli*. The most common combination of antimicrobial resistance phenotype in Belgian CiproR-*E. coli* was ampicillin-ciprofloxacin–trimethoprim/sulfamethoxazole (38.9% and 28.7% of the isolates from broilers and pigs respectively) and ampicillin-amoxicillin/clavulanic acid-ciprofloxacin-trimethoprim/sulfamethoxazole in Dutch CiproR-*E. coli* isolates from broilers (42.5% of the isolates) and pigs (84.6% of the isolates).

The percentage of multidrug-resistant (MDR) *E. coli* was high in pigs and broilers in both countries (Table 3). Resistance levels of the strains varied. In some farms, resistance to eight antibiotic classes was observed, while in other farms, bacteria resistant to only one class were isolated (Supplementary Figure S2).

## 3. Discussion

This study compared antibiotic use and resistance in broiler and pig farms in two bordering regions with comparable farming practices using similar data collection and analytical methods [15]. Carbapenems are not authorized for use in animals in the EU [8], and these drugs were not used in the year before sampling in the studied farms. 

Carbapenem-resistant *E. coli* were not detected in samples from broilers and pigs in Belgium and the Netherlands. However, among samples from Belgian broilers, 85% and 88% were positive for ESBL-*E. coli* and CiproR-*E. coli*, respectively, whereas among samples from Belgian pigs, 37% and 33% were positive for ESBL-*E. coli* and CiproR-*E. coli*, respectively. High rates of ESBL-*E. coli* have been previously reported in Belgian broilers (45%) [23] and in pigs (>70%) [24]. Similarly, high rates of CiproR-*E. coli* from Belgian broilers have been previously reported in 2015 (>60%) [19], 2017 [25] and 2018 (>50%) [8]. The rates of ESBL-*E. coli* and CiproR-*E. coli* were lower in samples from Dutch broilers (27% and 82% respectively) and pigs (4.0% and 11%, respectively). Similar rates of ESBL/AmpC-producing *E. coli* in feces of Dutch broilers (i.e., 33%) and slaughter pigs (i.e., 11%) were reported in 2017 by the Dutch monitoring system, MARAN [26]. However, this MARAN survey of 2017 reported only 34% of CiproR-*E. coli* from fecal samples of broilers and 2% of the *E. coli* from pig fecal samples [26]. The higher rates of CiproR-*E. coli* in our study might be explained by differences in farm selection. Indeed, in the MARAN survey, a stratified random sampling strategy was used, whereas in our study, farms with a history of high antibiotic use were selected. Finally, we also showed that the rates of ESBL-*E. coli* co-resistant to ciprofloxacin was higher in Belgium (33% in broilers and 17% in pigs) compared to the Netherlands (13% in broilers and 0% in pigs). 

The veterinary sales of critically important antibiotics to human health care (3rd and 4th generation cephalosporins and fluoroquinolones) fell sharply in both Belgium and the Netherlands [16,18,26]. However, the restriction of the these antibiotics for veterinary use was implemented earlier in the Netherlands (in 2013) [27] than in Belgium (in 2016) [28]. These differences in antibiotic policy between Belgium and the Netherlands could explain the observed differences of ESBL-*E. coli* and CiproR-*E. coli.* The high rates of CiproR-*E. coli* in samples from Dutch broilers could be explained by the higher use of flumequine and fluoroquinolones [29] in most Dutch farms compared with Belgian farms.

Several studies have shown an association between antibiotic use and resistance at national level [30] and animal level [2]. However, we could not demonstrate a clear link between the level of antibiotic use on farms during the year preceding sampling and the rates of antibiotic-resistant *E. coli* from fecal samples per farm. Our study was not powered to establish relationships between these variables. Moreover, we selected farms with a higher than average antibiotic use, which introduced a bias. Several other factors account for emergence of antibiotic resistance, not necessarily related to antibiotic use on farms during the year preceding sampling, such as antibiotic use in earlier stages of the production chain and the farm environment. Indeed, high rates of antibiotic-resistant *E. coli* in the studied farms could also be due to the use of antibiotics in the primary breeding companies at the top of the pyramid in the broiler production systems. The Netherlands Veterinary Medicines Authority (SDa) reported high fluoroquinolone use in poultry farming subsectors, mainly due to the use in broiler parent and grandparent stock [31]. Dierikx et al., (2013) showed the presence of ESBL/AmpC- producing *E. coli* isolates in the grandparent stock, one-day-old parent stock chicks and broiler chickens [32]. The same study also reported the use of enrofloxacin in the grandparent stock to prevent mortality from *E. coli* infection. Contamination of consecutive flocks could be caused by recirculation of resistant strains present in the farm environment [32]. High antibiotic resistance rates in fecal samples may also be explained by exposure to cumulated, resistance genes in litter or dust, or by additional introduction from non-poultry sources, such as water or other animals present on the farms [33,34].

Our study has several methodological specificities and limitations. We estimated the percentage of resistant samples based on selective culturing of bacteria followed by phenotypic antibiotic resistance determination. Hence, a sample is considered positive when resistant Enterobacteriaceae are present in the sample. This method is different from studies where estimation is based on randomly isolated resistant bacteria as a percentage of a population of bacteria. In addition, the number of samples investigated for presence of CiproR-*E. coli* was reduced to six samples per farm in six Belgian broiler farms (ID 9–15) and five Dutch broiler farms (ID 10–14), which might lead to a less accurate estimation of the presence of CiproR-*E. coli* in these farms. Antibiotic susceptibility testing was performed separately for Belgian and Dutch isolates with two distinct methods (disc diffusion and broth dilution). However, both methods provide a qualitative assessment of the susceptibility or resistance of the isolates and should not impact the resistance rates in each country. Finally, because of low prevalence of enterobacterial species other than *E. coli* (8.6%), these were excluded from the analysis. 

## 4. Materials and Methods

### 4.1. Study Design, Farm Selection and Farm Characteristics

In this cross-sectional study, 60 farms were included in Belgium and the Netherlands, comprising 29 conventional broiler farms (Belgium: N = 15, the Netherlands: N = 14) and 31 multiplier pig farms (Belgium: N = 15, the Netherlands: N = 16). Farms were recruited between March 2017 and July 2017. The farms were required to be located in either Flanders (Belgium) and the three southern provinces of the Netherlands and participation was voluntary. The farms were included based on the relative level of antibiotic use; meaning that antibiotic use was higher than average compared to the national benchmark value in the respective countries as described previously [22]. The farm characteristics are summarized in Supplementary Tables S1 and S2 and are described by Caekebeke et al., (2020) [22].

### 4.2. Antibiotic Use

Antibiotic use was calculated from registration documents provided by national quality assurance organizations, the farmers or farm veterinarians. Antibiotic use was quantified as the TI per 100 days for pigs and per production round for broilers [35] as described by Caekebeke et al., (2020) [22]. Total TI (referred to as TI tot) was defined as the average TI per round (broilers) or per 100 days (pigs) in the year preceding sampling. Likewise, TI of beta-lactams (phenoxymethylpenicillin, procaine benzylpenicillin, ampicillin, amoxicillin, cefalexin, ceftiofur, cefquinome) and TI of fluoroquinolones (enrofloxacin, flumequine) is hereafter referred to as TI BL and TI FQ (Supplementary Tables S1 and S2).

### 4.3. Collection of Fecal Samples 

The sampling period lasted six months, from the end of September 2017 to the beginning of April 2018, with the specific dates of sampling shown in Supplementary Tables S1 and S2. Samples were collected in a stratified-random sampling design based on the number of available units (broiler houses or rooms with weaned pigs). Within a farm, samples were collected from different units when more than one unit was present to take into account intra-farm variability. A maximum of three units were sampled per farm. The collection of 30 fecal samples per farm was aimed, evenly distributed over the selected units resulting in a total of 1800 samples. Fresh fecal droppings were collected from the stable floors using a nylon-flocked swab with 2 mL Cary-Blair transport medium (FecalSwab^TM^, Copan Italy, Brescia, Italy).

Broilers were sampled at approximately 35 days of age and weaned pigs between 8 and 10 weeks of age. After testing the first broiler farms, the observed high percentage of samples with CiproR-*E. coli* allowed for the reduction to six samples per farm in six remaining Belgian broiler farms (ID 9–15) and five remaining Dutch broiler farms (ID 10–14) for reasons of costs and workload in the laboratory (Supplementary Table S1).

### 4.4. Microbiological Methods

Fecal samples were submitted for microbiological analysis as described by Kluytmans-van den Bergh et al., (2019) [36]. A non-selective enrichment in tryptic soy broth (TSB) (Copan Italy, Brescia, Italy) was followed by subculturing 10 μL of TSB on selective agars, namely CHROMID^®^ ESBL, CHROMID^®^ CARBA, CHROMID^®^ OXA-48 (bioMérieux, Marcy l’Etoile, France) and MacConkey agar (Oxoid, Thermo Fisher Scientific, Basingstoke, UK) supplemented with 2 mg/L ciprofloxacin (Sigma-Aldrich, Saint Louis, MO, USA). TSB and plates were incubated for 18–24 h at 35–37 °C under aerobic conditions. Distinctive colonies on the agar plates were selected for species identification with MALDI Biotyper IVD (Bruker, MA, USA) for Belgian isolates and VITEK^®^ MS (bioMérieux, Marcy l’Etoile, France) for Dutch isolates. 

Subsequently, antibiotic susceptibility testing was performed on all isolates identified as *E. coli* (between one and five distinct *E. coli* per sample). Antibiotic susceptibility testing was performed in two laboratories with a separate panel for antibiotic susceptibility testing. For isolates originating from Dutch farms, minimum inhibitory concentrations for the following antibiotics were determined by broth microdilution VITEK^®^ 2 (N344) (bioMérieux, Marcy l’Etoile, France): ampicillin, amoxicillin–clavulanic acid, piperacillin–tazobactam, cefoxitin, cefuroxime, ceftazidime, cefotaxime, ciprofloxacin, gentamicin, meropenem, trimethoprim–sulfamethoxazole (1:19) and fosfomycin. Antimicrobial susceptibility of Belgian isolates was tested for ampicillin (10 μg), amoxicillin–clavulanic acid (20/10 μg), piperacillin–tazobactam (30/6 μg), cefoxitin (30 μg) and cefuroxime (30 μg), ceftriaxone (30 μg) and ceftazidime (10 μg), ciprofloxacin (5 μg), meropenem (10 μg), amikacin (30 μg), trimethoprim–sulfamethoxazole (1.25/23.75 μg) and fosfomycin (200 μg) using disk diffusion (Rosco, Taastrup, Denmark). Individual isolates were classified as susceptible, intermediate or resistant according to the EUCAST (v8.1) clinical breakpoints [37]. The combination disk diffusion method was used to confirm the presence of ESBL-*E. coli*. For this, the antibacterial activity of cefepime (30 μg), cefotaxime (30 μg) and ceftazidime (30 μg) with and without clavulanic acid (10 μg, Rosco, Taastrup, Denmark) was assessed. The reduction of bacterial growth (reduction of inhibition zone ≥ 5 mm) when the cephalosporin is combined with clavulanic acid was considered indicative for ESBL production [38].

### 4.5. Data Analysis

Statistics were performed for broilers and pigs separately in statistical program R version 4.0.2. [39]. The odds of a positive sample was analyzed using a mixed effects logistic regression model [40] with country and categorized antibiotic use as explanatory variables and with the number of positive samples from the total samples as outcome variable. Quantity of antibiotic use in the year preceding sampling was categorized in quartiles of treatment incidence (TI) of total antibiotic use and beta-lactams and use or no use of fluoroquinolone antibiotics (Supplementary Table S3). Farm was added to the model to account for the correlation between the sample results within a farm. The odds ratio (OR) was calculated with 95% confidence interval.

The percentage of samples with resistant bacteria was calculated as the number of positive samples divided by the total number of samples. MDR was determined based on the antimicrobial categories as described by Magiorakos et al., (2012) [41]. MDR was defined as resistance to at least one agent in at least three antimicrobial categories.

## 5. Conclusions

In conclusion, we provide unified information on the quantity of antibiotic use and presence of antibiotic resistance at the level of the farm in two neighboring countries with different antibiotic policies. Based on comparable and harmonized data on antibiotic use and resistance, we demonstrated clear differences in antibiotic resistance in farms with a history of high antibiotic use between the border regions of Belgium and the Netherlands. Harmonized data on antibiotic use and resistance leads to improved comparability of results and could lead to better implementation of stewardship actions. The study provides opportunities to create awareness among farmers, veterinarians and stakeholders of alarming rates of antibiotic resistance.

## Figures and Tables

**Table 1:** Distribution of farm level percentage of positive samples for ESBL-*E. coli* and CiproR-*E. coli* in Belgian and Dutch broiler and pig farms with estimated odds ratio for a positive sample.

	Broiler							
		Number of Samples	Percentage Positive Samples (%)	Number of Positive Farms	Min–Max within Farm Percentage (Percentage Positive Samples per Farm)	Median Percentage (%)	Interquartile Range (%)	OR NL vs. BE (95% CI)
ESBL-*E. coli*	BE	399	85	15/15	50–100	85	80–93	1 (reference)
	NL	380	27	10/14	0–100	15	0.83–43	0.007 (0.001–0.048)
CiproR-*E. coli*	BE	283	88	15/15	71–100	90	85–100	1 (reference)
	NL	303	82	14/14	33–100	90	72–97	0.60 (0.24–1.47)
	**Pig**							
		**Number of Samples**	**Percentage Positive Samples** **(%)**	**Number of Positive Farms**	**Min–Max within Farm Percentage (Percentage Positive Samples per Farm)**	**Median** **Percentage** **(%)**	**Interquartile Range** **(%)**	**OR NL vs. BE** **(95% CI)**
ESBL-*E. coli*	BE	399	37	13/15	0–95	28	10–54	1 (reference)
	NL	418	4.0	2/16	0–27	0	0–0	0.004 (0–0.042)
CiproR-*E. coli*	BE	399	33	14/15	0–95	23	13–51	1 (reference)
	NL	328	11	2/13	0–100	0	0–0	0.006 (0–0.098)

BE = Belgium, NL = the Netherlands, OR = odds ratio, CI = confidence interval.

**Table 2:** Associations between antibiotic use and prevalence of resistant samples in broiler and pig farms using a mixed effects logistic regression model. The model showed no association of any level of antibiotic use with prevalence. The quantity of antibiotic use in the year preceding sampling was categorized in quartiles of treatment incidence (TI) of total antibiotic use and beta-lactam use and use or no use of fluoroquinolone antibiotics.

	ESBL-*E. coli*			CiproR-*E. coli*		
**Broiler**	**Category total TI**	**OR**	**95% CI**	**Category total TI**	**OR**	**95% CI**
	Belgium, total TI < 2.9	1 (reference)		Belgium, total TI < 2.9	1 (reference)	
	The Netherlands	0.02	0–0.09	The Netherlands	0.46	0.19–1.07
	Total TI 2nd quartile [2.9– <6.2]	0.80	0.07–8.03	Total TI 2nd quartile [2.9– <6.2]	0.33	0.10–0.95
	Total TI 3rd quartile [6.2– <12.2]	1.20	0.1–12.22	Total TI 3rd quartile [6.2– <12.2]	0.40	0.11–1.24
	Total TI 4th quartile [12.2– <28]	0.95	0.08–11.54	Total TI 4th quartile [12.2– <28]	0.31	0.09–0.98
	**Category TI beta-lactam**	**OR**	**95% CI**	**Category TI fluoroquinolone**	**OR**	**95% CI**
	Belgium, TI_BL < 1.2	1 (reference)		Belgium, no fluroquinolone use	1 (reference)	
	The Netherlands	0.02	0–0.11	The Netherlands	0.45	0.16–1.22
	TI beta-lactam 2nd quartile [1.2– <3.4]	0.28	0.02–3.30	Fluoroquinolone use	1.69	0.63–4.77
	TI beta-lactam 3rd quartile [3.4– <7.4]	0.27	0.03–2.28			
	TI beta-lactam 4th quartile [7.4– <16]	0.33	0.03–2.81			
	**ESBL-*E. coli***			**CiproR-*E. coli***		
**Pig**	**Category total TI**	**OR**	**95% CI**	**Category total TI**	**OR**	**95% CI**
	Belgium, total TI < 12.9	1 (reference)		Belgium, total TI < 12.9	1 (reference)	
	The Netherlands	0.01	0.00–0.11	The Netherlands	0.01	0–0.05
	Total TI 2nd quartile [12.9– <23.2]	0.04	0.00–1.77	Total TI 2nd quartile [12.9– <23.2]	0.07	0–1.61
	Total TI 3rd quartile [23.2– <44]	0.63	0.03–15.90	Total TI 3rd quartile [23.2– <44]	0.48	0.03–5.04
	Total TI 4th quartile [44– <82]	0.20	0.01–7.40	Total TI 4th quartile [44– <82]	0.10	0.01–1.14
	**Category TI beta-lactam**	**OR**	**95% CI**	**Category TI fluoroquinolone**	**OR**	**95% CI**
	Belgium, TI beta-lactam < 3.2	1 (reference)		no fluoroquinolone use		
	The Netherlands	0	0–0.03			
	TI beta-lactam 2nd quartile [3.2– <12.1]	6.68	0.34–350.81			
	TI beta-lactam 3rd quartile [12.1– <22.7]	0.47	0.01–27.10			
	TI beta-lactam 4th quartile [22.7– <54]	0.22	0.00–9.93			

TI = treatment incidence, OR = odds ratio, CI = confidence interval.

**Table 3:** Multidrug resistance in *E. coli* from broilers and pigs. Number of isolates tested (N) and the percentage (%) of MDR isolates. A total of 12 antibiotic agents were included per country, namely ampicillin, amoxicillin–clavulanic acid, piperacillin–tazobactam, cefoxitin, cefuroxime, ceftriaxone (Belgium)/cefotaxime (the Netherlands), ceftazidime, meropenem, amikacin (Belgium)/gentamycin (the Netherlands), ciprofloxacin, fosfomycin and trimethoprim–sulfamethoxazole.

		ESBL-*E. coli*		CiproR-*E. coli*	
		N	% MDR ^A^	N	% MDR
Broiler	Belgium	523	89.7	303	77.2
	The Netherlands	143	68.5	301	75.9
Pig	Belgium	201	99.5	164	73.8
	The Netherlands	16	100	39	100

MDR, multidrug-resistant; ^A^ MDR: resistant to at least one agent in at least three antimicrobial categories.

## Data Availability

The datasets presented in this article are not readily available because the project management needs to give its approval whether databases can be shared. Requests to access the datasets should be directed to i41health@amphia.nl.

## Supplementary Materials

The following are available online at https://www.mdpi.com/article/10.3390/antibiotics10080945/s1, Table S1: Farm characteristics and antibiotic use in terms of treatment incidence (TI) in the broiler farms, Table S2: Farm characteristics and antibiotic use in terms of treatment incidence (TI) in pig farms, Table S3: Categories of the quantity of antibiotic use in the year preceding sampling, presented as the quartiles of treatment incidence (TI) of total antibiotic use and beta-lactam use and use or no use of fluoroquinolone antibiotics, Figure S1: Percentage of samples positive for ESBL-*E. coli* (a) and CiproR-*E. coli* (b) per unit for Belgian and Dutch pig and broiler farms, Figure S2: Percentage of isolates that show antibiotic resistance to a number (1–8) of antibiotic classes (colors) per farm (*x*-axis) in ESBL-*E. coli* and CiproR-*E. coli* isolates from broiler chickens (a) and pigs (b) in Belgium (BE) and the Netherlands (NL).
