# Supplementary material for: ESBL-Producing, Carbapenem- and Ciprofloxacin-Resistant Escherichia coli in Belgian and Dutch Broiler and Pig Farms: A Cross-Sectional and Cross-Border Study
Source: Antibiotics (Basel). 2021 Aug 4;10(8):945. doi: 10.3390/antibiotics10080945 (PMC8388939; doi:10.3390/antibiotics10080945)
Supplement: Supplementary file 1 [file antibiotics-10-00945-s001.zip › antibiotics-1313372-supplementary.pdf]

Supplementary Materials

Table S1. Farm characteristics and antibiotic use in terms of treatment incidence (TI) in the broiler farms.

Belgium								The Netherlands							
**Farm ID**	**Date of Sampling**	**Total Number of Broilers**	**Number of Units**	**Number of Rounds Per Year**	**TI tot**	**TI BL**	**TI FQ**	**Farm ID**	**Date of Sampling**	**Total Number of Broilers**	**Number of Units**	**Number of Rounds Per Year**	**TI tot**	**TI BL**	**TI FQ**
**1**	25/09/’17	180,000	4	6	7.44	5.96	0	**1**	27/09/’17	41,500	1	7	11.69	9	1
**2**	26/09/’17	50,000	1	5	2.37	0	0	**2**	09/10/’17	150,000	6	7.5	2.55	1.26	0.36
**3**	03/10/'17	125,000	4	6.5	18.13	13.75	0	**3**	16/10/’ 17	107,000	4	7.5	2.26	0	0.87
**4**	09/10/’17	79,500	3	6-7	17.49	14.17	0	**4**	06/11/’17	91,000	3	7.5	1.75	0.98	0.74
**5**	11/10/’17	85,000	3	7.4	4.32	2.99	0.03	**5**	24/10/’17	51,000	3	6.5	6.08	4.5	0.96
**6**	18/10/’17	90,000	3	6.5	12.57	8.26	0	**6**	19/10/’17	490,000	10	7.5	23.9	15.75	1.5
**7**	30/10/’17	130,000	4	6.5	12.13	7.23	0	**7**	10/11/’17	140,000	4	7	2.94	1.22	0.63
**8**	30/10/’17	87,000	2	7.5	14.42	0	1.33	**8**	13/11/’17	70,000	2	7	4	3.45	0
**9**	09/11/’17	82,000	3	7.2	5.33	0.36	0	**9**	20/11/’ 17	77,700	2	7.5	1.88	1.59	0.06
**10**	21/11/’17	84,000	2	7.5	8.95	5.45	0	**10**	14/02/’18		5				
**11**	02/02/’18	75,000	3	7	6.27	1.17	0	**11**	15/02/’18	63,000	2	7	8.79	2.95	0.2
**12**	05/02/’18	60,000	2	7.5	27.51	15.62	0.14	**12**	14/02/’ 18	23,400	2	6.5	9.63	7.93	0
**13**	19/02/’18	85,000	3	7.8	2.84	0.46	0	**13**	14/03/’18	50,000	2	7.4	5.53	4.67	0
**14**	05/03/’18	53,000	2	7	4.85	3.54	0	**14**	26/03/’18	165,000	4	7.4	2.38	1.87	0.49
**15**	06/04/’18	85,000	2	7	14.68	3.41	0	**15**	NA						

TI tot = average TI per round in the year preceding sampling; TI BL = TI of beta-lactams in the year preceding sampling; TI FQ = TI of fluoroquinolones in the year preceding sampling.

Table S2. Farm characteristics and antibiotic use in terms of treatment incidence (TI) in pig farms.

Belgium						The Netherlands					
**Farm ID**	**Date of Sampling**	**Number of Weaned Pigs**	**TI tot**	**TI BL**	**TI FQ**	**Farm ID**	**Date of Sampling**	**Number of Weaned Pigs**	**TI tot**	**TI BL**	**TI FQ**
**1**	16/11/'17	1,404	75.33	38.77	0	**1**	31/01/'18	1,700	13.66	9.36	0
**2**	24/10/'17	725	60.32	41.03	0	**2**	07/02/'18	1,300	21.56	14.71	0
**3**	27/10/'17	6,000	52.81	27.34	0	**3**	23/02/'18	1,400	7.19	0.67	0
**4**	08/11/'17	2,220	25.9	12.09	0	**4**	17/10/'17	936	12.94	7.26	0
**5**	08/11/'17	688	6.07	1.36	0	**5**	27/11/'17	3,600	34.33	3	0
**6**	14/11/'17	1,275	45.57	15.3	0	**6**	16/11/'17	2,800	12.02	12.02	0
**7**	16/11/'17	1,104	81.14	50.05	0	**7**	14/11/'17	2,400	24.51	10.12	0
**8**	22/11/'17	705	44.1	14.57	0	**8**	01/11/'17	1,824	8.4	3.23	0
**9**	23/11/'17	1,200	33.35	17.46	0	**9**	10/10/'17	8,000	37.79	22.71	0
**10**	23/01/'18	2,100	71.81	23.1	0	**10**	17/01/'18	3,500	20.97	4.45	0
**11**	06/02/'18	200	23.25	23.25	0	**11**	22/01/'18	1,400	4.52	0.08	0
**12**	07/02/'18	1,855	24.71	13.41	0	**12**	15/01/'18	3,000	14.52	2.24	0
**13**	16/02/'18	1,400				**13**	20/02/'18	800	1.87	1.42	0
**14**	26/02/'18	750	74.56	53.95	0	**14**	06/02/'18	2,500	5.86	0.38	0
**15**	08/03/'18	827	21.32	7.42	0	**15**	08/02/'18	1,300	17.64	17.43	0
						**16**	11/10/'17				

TI tot = average TI per 100 days in the year preceding sampling; TI BL = TI of beta-lactams in the year preceding sampling; TI FQ = TI of fluoroquinolones in the year preceding sampling.

Table S3. Categories of the quantity of antibiotic use in the year preceding sampling, presented as quartiles of treatment incidence (TI) of total antibiotic use and beta-lactam use and use or no use of fluoroquinolone antibiotics.

		**Categories**		
	**Quartile**	**Total TI**	**TI beta-lactam**	**TI fluoroquinolone**
	1st	[0- <2.9]	[0- <1.2]	no use
**Broiler**	2nd	[2.9- <6.2]	[1.2- <3.4]	use
	3rd	[6.2- <12.2]	[3.4- <7.4]	
	4th	[12.2- <28]	[7.4- <16]	
		**Total TI**	**TI beta-lactam**	**TI fluoroquinolone**
	1st	[0- <12.9]	[0- <3.2]	no use
	2nd	[12.9- <23.2]	[3.2- <12.1]	
**Pig**	3rd	[23.2- <44]	[12.1- <22.7]	
	4th	[44- <82]	[22.7- <54]	

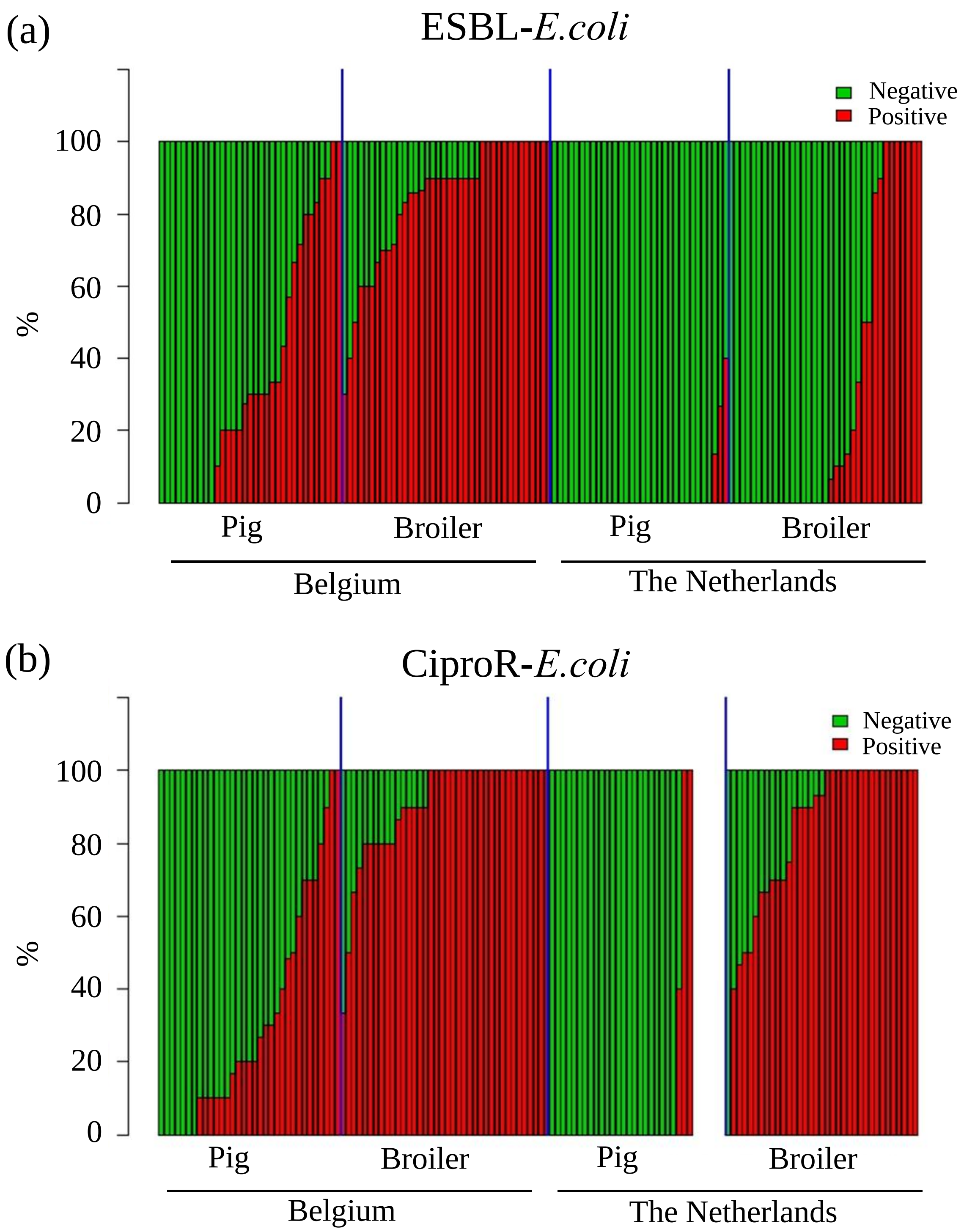

Figure S1. Percentage of samples positive for ESBL-*E. coli* (**a**) and CiproR-*E. coli* (**b**) per unit for Belgian and Dutch pig and broiler farms. BE = Belgium, NL = the Netherlands.

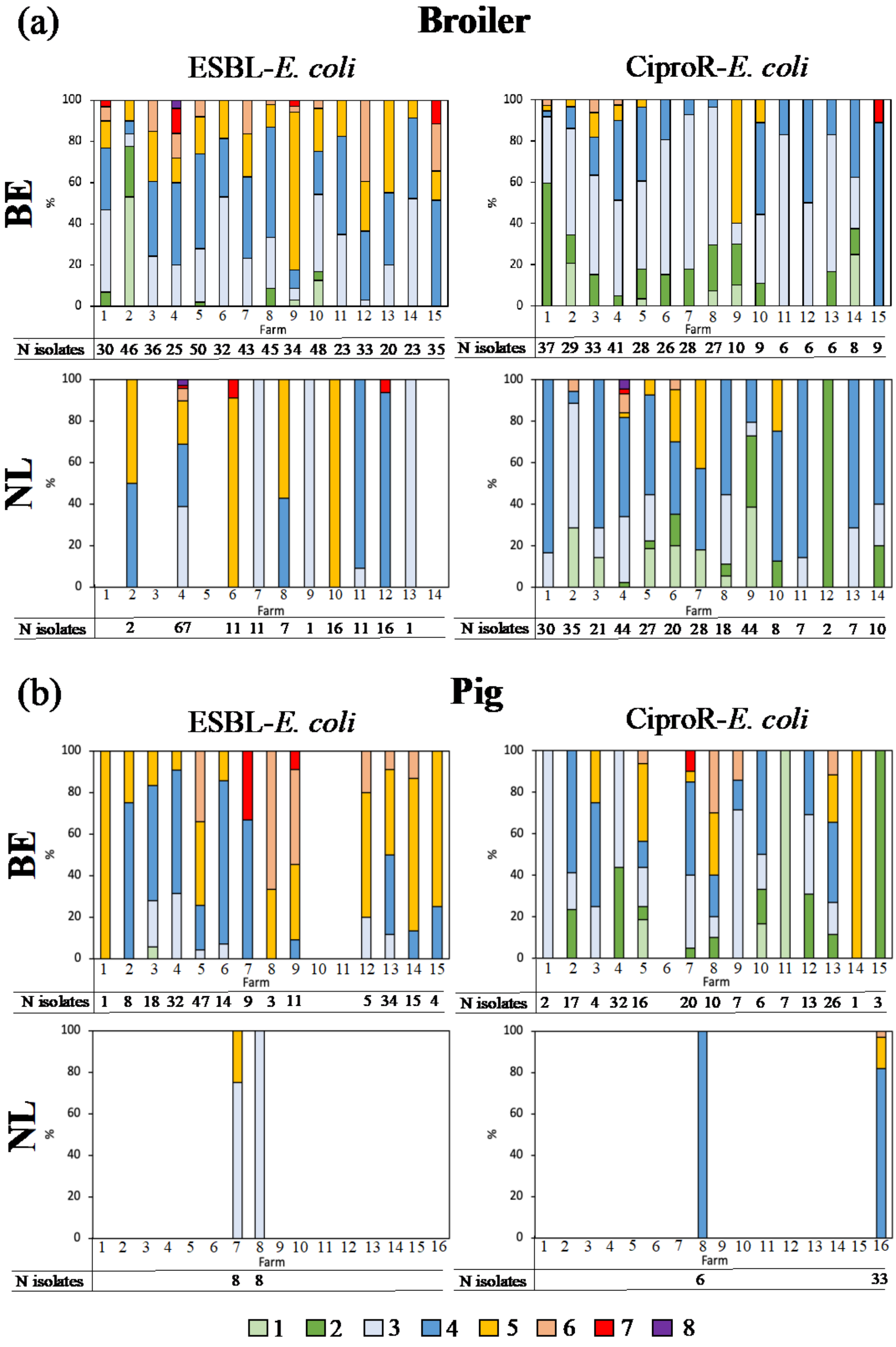

Figure S2. Percentage of isolates that show antibiotic resistance to a number (1-8) of antibiotic classes (colors) per farm (x-axis) in ESBL-*E. coli* and CiproR-*E. coli* isolates from broiler chickens (**a**) and pigs (**b**) in Belgium (BE) and the Netherlands (NL). N is the number of isolates evaluated. .
